# Knowledge and self-care practice among patients with hypertension in tertiary public hospitals of Addis Ababa, Ethiopia: A multicenter cross-sectional study

**DOI:** 10.1016/j.ijcrp.2024.200333

**Published:** 2024-09-11

**Authors:** Freweini Gebremeskel Gebresilase, Yohannes Ayalew Bekele, Ketema Bizuwork Gebremedhin, Boka Dugassa Tolera

**Affiliations:** a Addis Ababa University, College of Health Sciences, Tikur Anbessa Specialized Hospital, Ethiopia; b Addis Ababa University, College of Health Sciences, School of Nursing and Midwifery, Ethiopia

**Keywords:** Knowledge, Self-care practice, Hypertension

## Abstract

**Introduction:**

Globally, hypertension is the leading cause of death due to its related complications. Patients’ knowledge and self-care practice in hypertension is crucial for achieving optimal blood pressure control and prevention of related complications. This study aimed to evaluate the level of knowledge and self-care practice among hypertensive patients in Addis Ababa, Ethiopia.

**Methods:**

A facility-based cross-sectional study was conducted among 413 hypertensive patients using simple random sampling methods. A face-to-face interview was administered using a structured questionnaire. Data was analyzed using SPSS version 27.0. Frequency percentage, and mean were calculated. Multivariable logistic regression was used to identify the association between predictors and outcome variables.

**Results:**

Out of 413 respondents, 46.0 % (95 % CI: 40–50 %) and 40.9 % (95 % CI: 36–46 %) had poor knowledge and self-care practice respectively. Being married (AOR = 1.92, 95 % CI:1.19–3.06, *P* = 0.007)**,** higher education [AOR = 7.38 (95 % CI: 2.29–23.78), *P* < 0.001); family history (AOR = 3.68, 95 % CI: 2.28–5.94, *P* < 0.001); getting information from healthcare providers (AOR = 3.17, 95 % CI: 1.46–6.87, *P* = 0.003) were significantly associated with knowledge of hypertension. Being female (AOR: 0.62,95 % CI: 0.39–0.97, *P* = 0.033), owing sphygmomanometer (AOR: 4.41,95 % CI: 2.40–8.13, *P* < 0.001) were associated with self-care practice towards hypertension.

**Conclusion:**

The overall knowledge and self-care practice of respondents was low. Gender, marital status, educational level, family history, source of information, and owing sphygmomanometer were determinant factors. Improving patients’ awareness and self-care practice is essential for prevention and control of hypertension.

## Highlights

• Hypertension is the leading cause of death worldwide due to its high prevalence and related complications.
• Inadequate knowledge and self-care practice among patients with hypertension lead to cardiovascular complications.
• Knowledge and self-care practice of patients with hypertension are crucial in achieving optimal blood pressure control.
• The level of knowledge and self-care practice among hypertensive patients is low in developing nations.
• Expanding cardiovascular disease control initiatives is crucial to improve patient's knowledge and self-care practice.

## 1 Introduction

Hypertension is a chronic disease marked by increased arterial blood pressure and is the leading cause of mortality and mortality among cardiovascular diseases worldwide [1]. According to the WHO global report of 2023, over one billion people are living with hypertension worldwide [2]. The prevalence of hypertension is rising among developing countries, particularly in Africa, which is expected to rise to 216.8 million by 2030 [3]. The problem is due to myriad socio-demographics, individual lifestyles, and economic influences [4].

Hypertension poses great monetary challenges for individuals, families, and public health care systems in low and middle-income countries [5]. Ethiopia is one of the low-income countries that has the highest number of people living with hypertension in Africa [6]. A recent systematic review and meta-analysis conducted in Bahir Dar revealed the highest prevalence (20.63 %) of hypertension in Ethiopia [7]. This indicates that the burden of hypertension on society is enormous in terms of morbidity and mortality. The reason could be due to limited access to advanced healthcare systems, patients’ lack of awareness, and poor self-care behavior toward hypertension management and control.

The ultimate aim of hypertension management is to attain optimal blood pressure control and prevent related complications [8]. This means patients with hypertension may need to use various self-care practices including lifestyle modification, adherence to medications, eating a healthy diet, not smoking, regular monitoring of blood pressure level, and physical activity and reducing risk and related complications [9,10]. These helpful activities are thought to prevent the early occurrence of high blood pressure and related complications. However, in a resource constraint country like Ethiopia, it would be challenging to practice such kind of preventive measures because the level of self-care practice can be affected by several factors such as age, gender, having a family history of hypertension, lack of finances, poor medication adherence, educational level and lack of adequate knowledge about the disease [7,9,11].

Moreover, knowledge of hypertension is crucial in enhancing patients' adherence to self-care practices [12]. A previously published research has shown that having adequate knowledge about hypertension empowers good self-care practices, which ultimately impacts preventing hypertension-related complications [13]. Patients’ knowledge and awareness about hypertension not only promote self-management but also help the patient to effectively adhere to treatment and improve their quality of life [14]. Nevertheless, achieving optimal blood pressure control is difficult, and is a reason for related complications [15]. Therefore, good blood pressure control and the prevention of cardiovascular disease depend on the implementation of self-care management techniques [16].

Despite the pivotal role of patients’ knowledge and self-care practice in the management and control of hypertension, limited studies done in Ethiopia showed that the prevalence and incidence of hypertension are tremendously increasing [7,17].Hence, understanding the current level of knowledge and self-care behaviors among patients with hypertension may help to reduce the negative impact of hypertension by improving the quality of life [18], and may also point out disparities in developed and developing countries. Further, focusing on self-care management may lead to better treatment results and lower drug-related costs in a developing nation like Ethiopia where resources are scarce and treatment expenses are rising. Therefore, the present study aimed to evaluate the level of knowledge and self-care practice among patients with hypertension who were on follow-up at selected tertiary hospitals in Addis Ababa, Ethiopia.

## 2 Methods

### 2.1 Study design, settings, and populations

An institution-based descriptive cross-sectional study was conducted in Addis Ababa, the capital city of Ethiopia, located at an altitude of 2400 m above sea level [19]. The city has more than 42 hospitals, of which 13 are public. Even though all the hospitals in the city deliver generalized health care services, the four hospitals included in the current study deliver comprehensive hypertensive health care services. Further, these hospitals are prominent public hospitals in Addis Ababa, with equipped and separate chronic disease follow-up clinics for hypertension and diabetes. Namely, tertiary hospitals included in the current study were St Paul's Hospital Millennium Medical College (SPHMMC), Tikur Anbesa Specialized Hospital (TASH), Yekatit 12 Hospital, and Menelik II Referral and Comprehensive Hospital. The study was conducted from February15-March 15, 2022.

All adult patients with hypertension who attended the chronic clinic of the selected hospital were the source population and all adult patients who were on follow-up at the hypertensive clinic of the selected hospitals during data collection periods were the study population.

### 2.2 Eligibility criteria

This study included all adult patients with hypertension, who were on follow-up and available during the data collection period, and willing to provide consent to participate in the study. All patients with mental or neurologic disabilities, seriously ill, and having hearing problems were excluded from the study.

### 2.3 Sample size determination and recruitment procedures

**Fig. 1:**
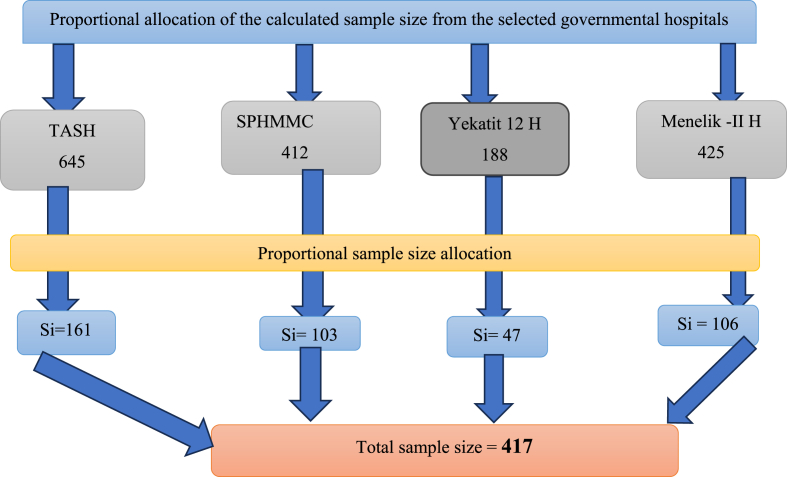
Schematic presentation of sampling procedure, N = 422; **Abbreviations:** TASH = Tikur Anbessa Specialized hospital; H = hospital, SPHMM= St. Paulos hospital and millennium medical college.

The sample size was determined using a single population proportion formula, n_o_ $=\frac{{\left(Z\right)}^{2}\left(P\right)\left(1-P\right)}{d^{2}}$ by considering previous estimates of the adequate knowledge level (56 %) among patient with hypertension [13]. Where: n_0_ = final sample size; (P*)* = proportion of patients who had good knowledge of hypertension; (1-P) = proportion of patients who don't have good self-practice; (d^2^) = 5 % margin of error; (*z*^2^) = 1.96 is standard normal deviation value corresponding to 95 % CI. By adding 10 % non-response rates, the total calculated sample size was 417. After obtaining an accurate representation of average monthly attendance from hypertensive clinics of selected hospitals, the study included 1670 hypertensive patients. Then we used a systematic random sampling method to recruit the study participants with a sampling interval of (K = 4). Accordingly, every fourth patient was asked to participate in the study. For those who did not consent to participate, we gave the chance to patient one to patient four. The below Fig. 1shows a schematic presentation of the sampling procedure.

### 2.4 Data collection tools and procedures

A structured face-to-face interviewer-administered questionnaire was adapted after an intensive review of related literature that focused on the knowledge and self-care practices of patients with hypertension [13,20]. The instrument was translated to the local language (Amharic) by language experts for ease of understanding and back to the original language to check its consistency. Before data collection, three clinical experts working in the cardiovascular department and 2 professors have validated the contents of the questionnaire. Then, the questionnaires were tested for internal consistency reliabilities giving a Cronbach's alpha coefficient = 0.89. The instrument contains three parts. Part I**:** Socio-demographic data; Part II: Knowledge about hypertension, and Part III was used to assess patients' self-care practices level regarding hypertension management. Some of the self-care practices questionnaire items were adapted from H-scales [21].The instrument was pretested in 5 % of the total samples in a healthcare institution other than the study setting (Zewditu Memorial Hospital) before the actual data collection period. Based on the analysis result of the pretest, the questionnaires were modified and prepared for a final version. On the goal and methodology of the study, as well as the data gathering methods, supervisors and data collectors received a one-day training.

The knowledge level of the study participants was measured by nine questions: 1) Does blood pressure inherited? 2) Is a blood pressure level above 140/90mmhg considered normal? 3) Is a blood pressure level less than 120/80 considered to be high? 4) Does smoking cigarettes have a negative effect on persons with hypertension? 5) Does drinking alcohol have a negative effect on persons with hypertension? 6) Does untreated cholesterol increase blood pressure? 7) Does untreated high blood pressure affect the heart 8) When to take pressure medicine? 9) Does the hypertensive patient should not be fed a spicy and salty diet? The knowledge level was measured based on the level of correct responses to the nine knowledge-measuring questions. The nine questions contained a total of nine highest points, with a correct response scoring one (1) point and a wrong response scoring zero (0) points. Finally, to categorize the knowledge level, we used modified Bloom's taxonomy cut-off point with a score of >75 % as good and ≤75 % as poor [22].

Self-care practice level of the study participants was measured by eight questions: 1) have you ever measured your cholesterol in your blood? 2) Do you ever stop taking your medicine if you're feeling better? 3) Do you have a home sphygmomanometer? 4) Do you regularly check your BP? 5) Do you consider food portions and choices whenever you have to eat food? 6) Do you perform physical exercise? 7) How often do you consult your doctor for HTN? 8) How often do you consult your cardiologist for a heart exam? Questions (1–6) were responded to as no/yes while questions (7 and 8) were responded to as “Sometimes and Always”. Finally, to categorize the self-care practice level, we used the median of the sum score of the self-care practice measuring questions, regarded as good for score ≥ median and poor for score < median. Socio-demographic characteristics, behavioral factors, family history of hypertension, source of information about hypertension, duration spent with the disease, and dietary approaches to prevent hypertension. The cutoff points for the knowledge and self-care practice level are summarized in Table 1.

**Table 1:** Cutoff points of knowledge and self-care practice towards hypertension (n = 413).

Knowledge level	
**> 7(75 %)**	Good
**≤ 7(75 %)**	Poor

**Self-care practice level**	
**≥ 15**	Good
**< 15**	Poor

### 2.5 Study variables

In this study, knowledge of hypertension and self-care practices were considered as dependent variables while socio-demographic factors such as age, sex, educational status, occupational status, marital status, clinical factors such as family history of hypertension, history of smoking, physical activities, source of information, duration of living with disease and availability of personal sphygmomanometer at home were considered as independent factors.

### 2.6 Operational definitions

We used the cut-off point as a baseline for the decision and interpretation of our result. Accordingly, the study variables were defined as follows:

Good knowledge: Participants who scored above the cut-off point on the close-ended knowledge questions regarding hypertension were considered as knowledgeable. Otherwise, they were considered to have poor knowledge [13].

Good self-care practice**:** The level of participants' self-care activities who scored above the mean score on the close-ended questions of self-care practice regarding hypertension management. Otherwise, they were considered as having poor self-care practices towards hypertension [13,23].

### 2.7 Ethical approval

The Institutional Review Board (IRB) of the Addis Ababa University College of Health Science granted the study ethical approval. The study has been carried out in accordance with the declaration of Helsinki. An official letter was written to the selected governmental hospitals and permission was obtained from the respective hospitals. Participation in the study was entirely voluntary, and participants were free to leave at any moment, as noted in the participant information sheet. Prior to the collection of data, each research participant signed an informed consent form. All personal identifiers were omitted throughout data collection and the collected data were stored and kept in locked storage. Only the principal investigator had access to use data.

### 2.8 Statistical analysis

The data were examined, cleansed, and imported into the Epi-data 3.1 version. Data was exported to the SPSS version 27.0 version for analysis. The participant characteristics were described using descriptive statistics such as percentage, frequency, and mean value. The chi-square test was used to determine the relationship between dependent and independent variables. Bivariable logistic regression analysis was performed to determine the crude relationship of the selected explanatory variable with dependent variables. Variables with a P-value of less than 0.25 in the bivariable logistic regression were fitted to the multivariable binary logistic regression model. Variables with a P < 0.05 were considered as a cut point for statistical significance. The association was reported using an adjusted odd ratio (AOR) with a 95 % CI. The Hosmer and Lemeshow goodness of test was used to examine the fit of the model. The final fitted model was attested and no multicollinearity was observed.

## 3 Results

### 3.1 Socio-demographic and background characteristics of the participants

In this study, 413 patients with hypertension were included giving a response rate of 99 %. The majority 89.8 %(n = 371) of the study participants were aged ≥40 years old. The mean age of the study participants was 57.2 ± 13.4 years. More than half, 58.6 % (n = 243), 53.3 % (n = 220), 58.8 %(n = 243), and 54.5 % (n = 225) of the study participants were female in gender, married in their marital status, attended secondary and above in their education, and earning less than or equal to 10,000Ethiopian birr in monthly income respectively. Furthermore, about 42.6 %(n = 176), 64.2 %(n = 265), and 53.5 % (n = 221) of the study participants were employed in their occupation, lived with the disease for over five years, and had no history of hypertension in their family respectively.

All of the study participants were aware of the disease at the first instant of their diagnosis of hypertension. The majority of the study participants' sources of information about the disease were health care professionals 81.8 %(n = 338), followed by mass media 11.6 % (n = 48) like television and radio, and the internet and broacher 6.5 % (n = 27) about the disease. Further, a significant proportion of 85.5 % (n = 353) and 53.3 % (n = 220) of the study participants had no sphygmomanometers in their homes and didn't engage in physical exercise as preventive behavior of the disease respectively (Table 2).

**Table 2:** Socio-demographic, and background characteristics of study participants at selected tertiary hospitals of Addis Ababa, Ethiopia (n = 413).

Variables	Frequency (%)	Percentage (%)	Mean (SD)
**Age in Years**			57.2 ± 13.4
**<40**	42	10.3	
**≥40**	371	89.8	
**Sex**			
**Male**	171	41.4	
**Female**	242	58.6	
**Marital status**			
**Unmarried**	193	46.7	
**Married**	220	53.3	
**Educational status**			
**Illiterate**	27	6.5	
**Primary**	143	34.6	
**Secondary and higher education**	243	58.8	
**Monthly income**			
**≤1000ETB**	225	54.5	
**>1000ETB**	188	45.5	
**Occupation**			
**Employed**	176	42.6	
**Unemployed**	132	32.0	
**Pensioned**	105	25.4	
**Duration of living with hypertension**			
**< Five-year**	148	35.8	
**≥ Five-year**	265	64.2	
**History of hypertension in the family**			
**No**	192	46.5	
**Yes**	221	53.5	
**Source of information about HPT at the first instant**			
**Mass medias**	48	11.6	
**Health professionals**	338	81.8	
**Other: internet, broachers**	27	6.5	
**Availability of sphygmomanometer at home**			
**No**	353	85.5	
**Yes**	60	14.5	
**Engaged in physical Exercise**			
**No**	220	53.3	
**Yes**	193	46.7	

**Note: Marital status:** unmarried includes single, divorced, separated, and widowed; **Occupation**: unemployed includes housewives, merchants, and farmers. Abbreviations: ETB = Ethiopian Birr; HPT = Hypertension; TV = Television.

### 3.2 Knowledge and self-care practice level of the study participants

**Fig. 2:**
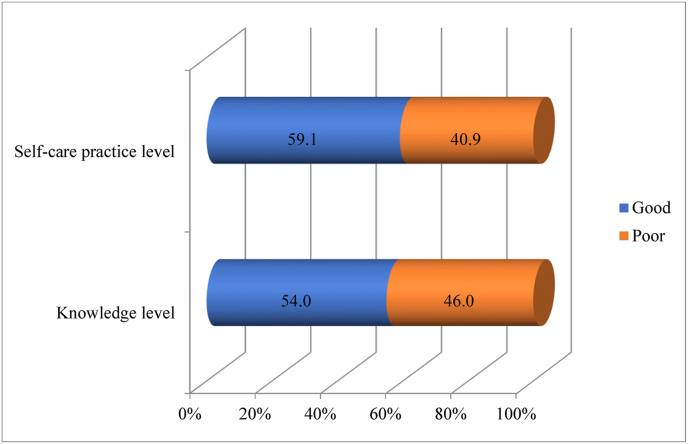
Knowledge and self-care practice level of study participants at selected tertiary hospitals in Addis Ababa, Ethiopia (n = 413).

The study revealed a significant 46.0 % (95 % CI: 40–50 %) and 40.9 % (95 % CI: 36–46 %) proportion of the study participants had poor levels of knowledge, and self-care practice respectively (Fig. 2) . As presented in Table 3, the higher poor knowledge about the disease was observed among younger age <40 years 57.1 %(n = 24), females in their gender 53.3 % (n = 129), unmarried in their marital status 54.4 % (n = 105) illiterate in their educational status 81.5 %(n = 22), earning monthly income less than 10,000 Ethiopian birr per month 57.3 % (n = 129), pensioned in their occupation 59 %(n = 62), living with the disease for less than five years 48 %(n = 71), had family history about the disease 62.0 %(n = 137) and use internet and printed materials as a source of information 70.4 %(n = 19) respectively. Further, a higher poor self-care practice level was observed among study participants who were female in their gender 46.7 %(n = 113), attended only a primary level in educational status 50.4 % (n = 71), living with the disease for more than and equal to five years 41.5 %(n = 110), and had no personal sphygmomanometer at their home 36.0 % (n = 127) respectively (Table 4)

**Table 3:** Factors associated with patients’ knowledge of hypertension at selected tertiary hospitals in Addis Ababa, Ethiopia (n = 413).

Variables	Knowledge level				
	Poor	Good	COR (95%CI)	AOR (95%CI)	P-value
Sex					
**Male**	61(35.7)	110(64.3)	1	1	1
**Female**	129(53.3)	113(46.7)	0.49(0.35–0.73) **	1.25(0.69–2.24)	0.460
Marital status					
**Unmarried**	105(54.4)	88(45.6)	1	1	1
**Married**	85(38.6)	135(61.4)	1.90(1.28–2.81) *	1.92(1.19–3.06) *	0.007
Educational status					
**Illiterate**	22(81.5)	5(18.5)	1	1	1
**Primary education**	96(67.1)	47(32.9)	2.15(0.77–6.05)	1.72(0.54–5.49	0.364
**SED & above**	72(29.6)	171(70.4)	10.45(3.81–28.67) **	7.38(2.29–23.78) **	<0.001
Monthly income					
**<10,000ETB**	131(58.2)	94(41.8)	1	1	1
**>10,000ETB**	59(31.4)	129(68.6)	3.05(2.03–4.57) **	1.37(0.81–2.32)	0.238
Occupation status					
**Employed**	77(43.7)	99(56.3)	1	1	1
**Unemployed**	51(38.6)	81(61.4)	1.24(0.78–1.96)	1.27(0.71, 2.27)	0.416
**Pensioned**	62(59.0)	43(41.0)	0.54(0.33–0.88) *	0.71(0.37–1.38)	0.311
Duration of living with hypertension					
**< 5years**	71(48.0)	77(52.0)	1	1	1
**≥ 5years**	119(44.9)	146(55.1)	1.13(0.76–1.69)	1.31(0.79–2.15)	0.284
Family history of hypertension					
**No**	53(27.6)	139(72.4)	1	1	1
**Yes**	137(62.0)	84(38.0)	4.28(2.82–6.49) **	3.68(2.28–5.94) **	<0.001
Source of information about hypertension at the first instant					
**Mass medias**	33(68.8)	15(31.2)	1	1	1
**Health professionals**	138(40.8)	200(59.2)	3.19(1.67–6.09) **	3.17(1.46–6.87) *	0.003
**Internet & printed materials**	19(70.4)	8(29.6)	0.93(0.33–2.59)	0.96(0.29–3.13)	0.942
Having a personal sphygmomanometer at home					
**No**	154(43.6)	199(56.4)	1	1	1
**Yes**	36(60.0)	24(40.0)	1.94(1.11–3.39) *	1.89(0.99–3.62)	0.054

**Note:** *Statistically Significant at p < 0.05, **Statistically Significant at p < 0.001, **Abbreviations**: ETB = Ethiopian Birr, SED = Secondary School and above, AOR = Adjusted Odds Ratio, COR= Crude Odds Ratio, CI= Confidence Interval.

**Table 4:** Factors associated with self-care practice among patients with hypertension at selected tertiary hospitals in Addis Ababa, Ethiopia (n = 413).

Variables	Self-care practice				
	Poor	Good	COR (95%CI)	AOR (95%CI)	P-value
Sex					
**Male**	56(32.7)	115(67.3)	1	1	1
**Female**	113(46.7)	129(53.3)	0.56(0.37–0.84) *	0.62(0.39–0.97) *	0.035
Educational status					
**Illiterate**	13(48.1)	14(51.9)	1	1	1
**Primary education**	72(50.3)	71(49.7)	0.92(0.40–2.09)	0.76(0.32–1.82)	0.539
**SED & above**	81(34.6)	159(65.4)	1.76(0.79–3.91)	1.24(0.52, 2.94)	0.626
Duration of living with hypertension					
**< 5years**	59(39.9)	89(60.1)	1	1	1
**≥ 5years**	110(41.5)	155(58.5)	0.93(0.62–1.41)	0.97(0.62–1.50)	0.875
Family history of hypertension					
**No**	67(34.9)	125(65.1)	1	1	1
**Yes**	103(46.4)	119(53.6)	1.60(1.08–2.38) *	1.35(0.88–2.07)	0.173
Availability of sphygmomanometer at home					
**No**	127(36.0)	226(64.0)	1	1	1
**Yes**	18(30.0)	42(70.0)	4.15(2.29–7.52) **	4.41(2.40, 8.13) **	<0.001

**Note:** *Statistically Significant at p < 0.05, **Statistically Significant at p < 0.001, **Abbreviations**: SED = Secondary School and above, AOR = Adjusted Odds Ratio, COR= Crude Odds Ratio, CI= Confidence Interval.

### 3.3 Factors associated with participants’ knowledge and self-care practice regarding hypertension management

The association of independent variables with the participant's knowledge level regarding hypertension management was observed using a logistic regression model. In multivariable logistic regression analysis, marital status being married [ AOR = 1.92 (95 % CI:1.19–3.06), *P* = 0.007]**,** educational status being attended secondary school and above level [AOR = 7.38 (95 % CI: 2.29–23.78), *P* < 0.001]; having a history of hypertension in the family [AOR = 3.68 (95 % CI: 2.28–5.94), *P* < 0.001]; being getting information about hypertension from health care providers [AOR = 3.17(95 % CI: 1.46–6.87), *P* = 0.003] were found more likely to have good knowledge towards hypertension (Table 3). Similarly, the association of independent variables with self-care practice regarding hypertension management was observed using a multivariable logistic regression model. Accordingly, being female [AOR: 0.62 (95 % CI: 0.39–0.97), *P* = 0.033] was less likely to have good self-care practices towards hypertension management. However, having a personal sphygmomanometer [AOR: 4.41 (95 % CI: 2.40–8.13), *P* < 0.001] was found more likely to have good self-care practice than those who have no personal sphygmomanometer at their home (Table 4).

## 4 Discussion

Globally, hypertension is a serious life-threatening disease due to its high prevalence and related complications [1]. Moreover, the prevalence of hypertension is steadily rising in nations with inadequate resources, and it is a significant public health concern [4,7].To evade complications related to hypertension and improve patients' quality of life, it is essential to have sufficient knowledge and practice superb self-care [12]. This study aimed to determine the level of knowledge and self-care behaviors among adult hypertension patients who were on follow-up at tertiary hospitals in Addis Ababa, Ethiopia. The findings of the current study revealed that participants’ knowledge and self-care practice regarding hypertension management was generally poor. Gender, marital status, educational level, family history of hypertension, getting information from healthcare providers, and owing sphygmomanometer were determinant factors associated with knowledge and self-care practice regarding hypertension management. Lack of adequate knowledge and poor self-care practice in the management and prevention of cardiovascular diseases like hypertension can negatively affect the health of individuals with the disease because hypertension is a life-threatening condition and might cause sudden death.

Knowledge of hypertension is crucial in enhancing patients’ adherence to self-care practices [12]. However, in the present study, about 46.0 % (95 % CI: 40–50 %) of the study participants had a poor level of knowledge regarding hypertension. This finding is in line with study findings in India (47.6 %) [24], and Northern Ethiopia(44.0 %) [13].In light of this finding, it is important to note that the management of high blood pressure will not be achieved and people living with hypertension either suffer because of a lack of adequate knowledge about the disease. But, our finding is better than the study reported from Burkina Faso (82.5 %) [25], and Vietnam (70.6 %) [26], lower than the study finding from Nigeria (94.4 %) [27]. Inconsistency between the findings could be due to the difference in socio-cultural variation, differences in living status of the study participants, sample size, study participants, demographic characteristics, and educational level of the study participants. This finding underscores the need to create awareness by providing health education through multiple media and social networking.

Self-care practices are essential for managing and preventing hypertension since they enhance the quality of life, and save medical costs [18],. However, the present study indicates only 40.9 % (95 % CI:36–46 %) of the participants had poor self-care practices. This finding is in agreement with previous study studies from Northwest Ethiopia (45.9 %) [28] and Southern Tunisia (45.2 %) [29], but our finding is better than the result of the study reported from Eastern Ethiopia (70.1 %) [30]. The possible reason could be due to differences in the study area, which means the current study was conducted in urban but the previous study was done in rural and semi-urban areas where the chance to get information and knowledge on self-care practice is relatively lower than those living in urban areas. In addition to this, lifestyle, cultural, and socio-economic features of the society varied across the communities in Ethiopia. The consequence of poor self-care practices for chronic disease management like uncontrolled hypertension is the rapid development of comorbidities such as diabetes mellitus [31], renal disease, and ectopic fat depots [32] which have a financial and social burden on their patients, families, and health care system. Therefore, providing continuous education and counseling patients regarding daily self-care practices such as lifestyle modifications, performing physical activities, and the benefit of medication adherence should be addressed by healthcare providers during follow-up visits.

This study demonstrated that married individuals had nearly 1.92 times higher odds of having a good level of knowledge about hypertension than those who were not married. A similar report from prior cross-sectional studies demonstrated that knowledge and awareness of hypertension control were higher in married people than in single [33], regardless of occupation, age, and ethnic group. Further, in the study conducted by Wang, X. et al.(2023), people living alone did not have good knowledge and self-care practices regarding hypertension control [34]. This might be because married individuals have a chance of better communication and discussion with their couple and family about the disease. Hence, it is expected that individuals who contact his/her spouse and family are more likely to be knowledgeable about the disease. Therefore, couple support is a key to improvements in knowledge and understanding of BP control.

In line with the previous studies conducted in Nigeria [27] and Burkina Faso [25], the results of the current study revealed that participants who attended secondary school and higher educational levels had 7.38 times higher odds of good knowledge of hypertension management compared to those who were illiterate. This signifies those participants who attended higher educational levels probably had a better understanding of self-care practices for managing their hypertension and related problems. Because higher-educated people can use different information sources than people with no formal education [27]. Consequently, when patients attended a higher level of education, they were more likely to have good knowledge of disease self-care behaviors, such as medication adherence, diet maintenance, physical exercise, and illness self-monitoring.

The substantial association between a family history of hypertension and a high level of knowledge regarding hypertension is another intriguing finding from this study. The results of this study showed that in comparison to individuals without a family history of hypertension, those with a history of hypertension were almost four times more likely to have adequate knowledge about the disease. This result is in line with earlier research from Northwest Ethiopia [20] and Japan [35], which reveals a positive and strong association between family history and a good knowledge of hypertension control. The possible explanation might be study participants with a family history of hypertension have better information related to the disease, and have an opportunity to get an education from their family.

The current study also revealed that study participants who received information from healthcare providers were nearly four times more likely to have a good level of knowledge about hypertension control compared to those who did not receive any information about hypertension. This finding is in line with the reports from Tunisia [29] and Thailand [36]. This could be due to the fact that the information provided to the patient by health professionals is one way, which can transform the information into knowledge, and trigger them to use it. As of a recent study conducted by Gebreziher, L.H. et al.(2024), less frequency of visiting clinicians was associated with a high prevalence and incidence of uncontrolled hypertension and related comorbidities [31]. This indicates that patients with chronic diseases like hypertension should have regular interactions with healthcare professionals which can help them to get crucial information about the management and control of hypertension.

Adherence to self-care activities is key to blood pressure control in patients diagnosed with hypertension [9]. However, the degree of self-care activities among individuals with hypertension is influenced by several socio-demographic factors. The findings of this study showed that females with hypertension were 0.61 times less likely to have good self-care practices compared to males with hypertension. This finding is supported by the study done in Southwest Ethiopia [37] and India [38]. The possible reason could be males were more likely to involve in physical exercise and engage himself in hard work than females. Additionally, as the finding of our study shows, males are better educated and have the awareness of better clinical follow-up than female patients. However, our finding is not similar to the study reported in China [39] where males have lower self-care practices than female hypertensive patients. The possible reason could be differences in culture lifestyle, and social support. In our study area, due to cultural norms, women are expected to spend their time at home taking care of their families' needs and preparing meals. Additionally, women may lack confidence, receive less family support for self-care, blame themselves more for their illness, and disregard their spouses' and children's needs.

Furthermore, having a personal sphygmomanometer at home was another factor that was found to be significantly associated with good self-care practices. Respondents who had a personal sphygmomanometer were five times more likely to engage in good self-care practices. This finding corresponds to prior research studies conducted in Northeast Ethiopia [40], Turkey [41] and Korea [42]. The association could be highlighted by pointing out that possibly, having a personal sphygmomanometer at home gives a chance to have regular monitoring of blood pressure levels, allowing patients to stay informed about their blood pressure status, which in turn may lead to better awareness of hypertension self-care practices by promoting lifestyle changes. Generally, hypertension is undoubtedly one of the fastest-growing public health problems worldwide. Improving the patients’ knowledge and self-care practice towards hypertension is crucial for the prevention of hypertension and related complications [2]. Additionally, it can serve as the starting point for lowering the burden on the healthcare system and accomplishing the sustainable development goal.

## 5 Strengths and weakness of the study

The study was conducted in Addis Ababa, where the hypertension burden is very high. Hence, the finding provides empirical evidence for the improvement of public health by developing important preventive strategies. Despite this, our study has some limitations. The study was cross-sectional and cannot determine a cause-effect relationship. On the other hand, using an interview-administered questionnaire may result in socially desirable bias on patients' knowledge and self-care practices. In addition to this, hospital-based studies cannot give an accurate result of knowledge and self-care practice of the community because some patients may not be available at the study institution during the study periods.

## 6 Conclusion

This study revealed that the level of knowledge and self-care practice among patients with hypertension was low. Gender, marital status, educational level, family history of hypertension, getting information from healthcare providers, and owing sphygmomanometer were determinant factors associated with the level of knowledge and self-care practice regarding hypertension control. Therefore, all concerned bodies should design a strategy to close the gap between knowledge and practice. Moreover, healthcare professionals should provide adequate information and education regarding self-care practices for the prevention and control of hypertension. Further studies including qualitative or large-scale prospective cohort studies should be needed to identify risk factors for hypertension among adults.

## Funding

The authors received no financial support for the research, authorship, & publication of this article.

## Data availability

The datasets used and analyzed during the current study are available upon request.

## Declaration of interest statements

The authors declared no financial interests or personal relationships that could have appeared to influence the work reported in this paper.

## CRediT authorship contribution statement

**Freweini Gebremeskel Gebresilase:** Writing – review & editing, Writing – original draft, Validation, Methodology, Formal analysis, Data curation, Conceptualization. **Yohannes Ayalew Bekele:** Writing – review & editing, Writing – original draft, Validation, Supervision, Software, Conceptualization. **Ketema Bizuwork Gebremedhin:** Writing – review & editing, Writing – original draft, Validation, Supervision, Software, Methodology, Data curation, Conceptualization. **Boka Dugassa Tolera:** Writing – review & editing, Writing – original draft, Validation, Supervision, Software, Project administration, Methodology, Investigation, Formal analysis, Data curation, Conceptualization.

## Declaration of generative AI and AI-assisted technologies in the writing process

During the preparation of this work, the authors didn't use any generative AI and AI-assisted technologies in the writing process.
